# Supplementary material for: The Experiences of Stakeholders Using Social Media as a Tool for Health Service Design and Quality Improvement: A Scoping Review
Source: Int J Environ Res Public Health. 2022 Nov 11;19(22):14851. doi: 10.3390/ijerph192214851 (PMC9690250; doi:10.3390/ijerph192214851)
Supplement: Supplementary file 1 [file ijerph-19-14851-s001.zip › ijerph-1977980-supplementary.pdf]

Supplementary Materials:

Table S1. Preferred Reporting Items for Systematic reviews and Meta-Analyses extension for Scoping Reviews (PRISMA-ScR) Checklist.

SECTION	ITEM	PRISMA-ScR CHECKLIST ITEM	REPORTED ON PAGE #
**TITLE**			
Title	1	Identify the report as a scoping review.	1
**ABSTRACT**			
Structured summary	2	Provide a structured summary that includes (as applicable): background, objectives, eligibility criteria, sources of evidence, charting methods, results, and conclusions that relate to the review questions and objectives.	1
**INTRODUCTION**			
Rationale	3	Describe the rationale for the review in the context of what is already known. Explain why the review questions/objectives lend themselves to a scoping review approach.	2-3
Objectives	4	Provide an explicit statement of the questions and objectives being addressed with reference to their key elements (e.g., population or participants, concepts, and context) or other relevant key elements used to conceptualize the review questions and/or objectives.	3
**METHODS**			
Protocol and registration	5	Indicate whether a review protocol exists; state if and where it can be accessed (e.g., a Web address); and if available, provide registration information, including the registration number.	3, weblink in reference list
Eligibility criteria	6	Specify characteristics of the sources of evidence used as eligibility criteria (e.g., years considered, language, and publication status), and provide a rationale.	4-6
Information sources*	7	Describe all information sources in the search (e.g., databases with dates of coverage and contact with authors to identify additional sources), as well as the date the most recent search was executed.	6-7
Search	8	Present the full electronic search strategy for at least 1 database, including any limits used, such that it could be repeated.	Appendix 2
Selection of sources of evidence†	9	State the process for selecting sources of evidence (i.e., screening and eligibility) included in the scoping review.	6-7
Data charting process‡	10	Describe the methods of charting data from the included sources of evidence (e.g., calibrated forms or forms that have been tested by the team before their use, and whether data charting was done independently or in duplicate) and any processes for obtaining and confirming data from investigators.	7, Appendix 3

SECTION	ITEM	PRISMA-ScR CHECKLIST ITEM	REPORTED ON PAGE #
Data items	11	List and define all variables for which data were sought and any assumptions and simplifications made.	4-8
Critical appraisal of individual sources of evidence§	12	If done, provide a rationale for conducting a critical appraisal of included sources of evidence; describe the methods used and how this information was used in any data synthesis (if appropriate).	Not undertaken – noted as limitation
Synthesis of results	13	Describe the methods of handling and summarizing the data that were charted.	7
**RESULTS**			
Selection of sources of evidence	14	Give numbers of sources of evidence screened, assessed for eligibility, and included in the review, with reasons for exclusions at each stage, ideally using a flow diagram.	Figure 1
Characteristics of sources of evidence	15	For each source of evidence, present characteristics for which data were charted and provide the citations.	Table 2, full citations in reference list
Critical appraisal within sources of evidence	16	If done, present data on critical appraisal of included sources of evidence (see item 12).	Not undertaken
Results of individual sources of evidence	17	For each included source of evidence, present the relevant data that were charted that relate to the review questions and objectives.	Table 2, Appendix 4
Synthesis of results	18	Summarize and/or present the charting results as they relate to the review questions and objectives.	8-26, Appendix 4
**DISCUSSION**			
Summary of evidence	19	Summarize the main results (including an overview of concepts, themes, and types of evidence available), link to the review questions and objectives, and consider the relevance to key groups.	17=33
Limitations	20	Discuss the limitations of the scoping review process.	31-32
Conclusions	21	Provide a general interpretation of the results with respect to the review questions and objectives, as well as potential implications and/or next steps.	33
**FUNDING**			
Funding	22	Describe sources of funding for the included sources of evidence, as well as sources of funding for the scoping review. Describe the role of the funders of the scoping review.	34

JBI = Joanna Briggs Institute; PRISMA-ScR = Preferred Reporting Items for Systematic reviews and Meta-Analyses extension for Scoping Reviews.

* Where *sources of evidence* (see second footnote) are compiled from, such as bibliographic databases, social media platforms, and Web sites.

† A more inclusive/heterogeneous term used to account for the different types of evidence or data sources (e.g., quantitative and/or qualitative research, expert opinion, and policy documents) that may be eligible in a scoping review as opposed to only studies. This is not to be confused with *information sources* (see first footnote).

‡ The frameworks by Arksey and O'Malley (6) and Levac and colleagues (7) and the JBI guidance (4, 5) refer to the process of data extraction in a scoping review as data charting.

§ The process of systematically examining research evidence to assess its validity, results, and relevance before using it to inform a decision. This term is used for items 12 and 19 instead of "risk of bias" (which is more

applicable to systematic reviews of interventions) to include and acknowledge the various sources of evidence that may be used in a scoping review (e.g., quantitative and/or qualitative research, expert opinion, and policy document).

From: Tricco AC, Lillie E, Zarin W, O'Brien KK, Colquhoun H, Levac D, et al. PRISMA Extension for Scoping Reviews (PRISMAScR): Checklist and Explanation. Ann Intern Med. 2018;169:467–473. doi: 10.7326/M18-0850.

Table S2. Example search strategies.

Example	Keyword and Title search	Date range
Medline OVID	(Consumer* OR patient* OR carer* OR public OR service user*) OR (Service provider* OR health professional* OR doctor* OR nurs* OR physiotherap* OR physical therap* OR allied health OR dietitian* OR speech patholog* OR medical OR pharmac* OR specialist* OR occupational therap* OR psycholog*) AND (social media OR facebook OR Twitter OR linkedin OR instagram OR snapchat OR blog OR tumblr OR myspace) AND (engag* OR participat* OR involv* OR partner* OR codesign* OR coprod* OR co-design* OR co-prod* OR quality improvement) AND (hospital* OR health service* OR medical service* OR policy OR policies)	2004 – current (16 August 2022)
Embase OVID	(Consumer* OR patient* OR carer* OR public OR service user*) OR (Service provider* OR health professional* OR doctor* OR nurs* OR physiotherap* OR physical therap* OR allied health OR dietitian* OR speech patholog* OR medical OR pharmac* OR specialist* OR occupational therap* OR psycholog*) AND (social media OR facebook OR Twitter OR linkedin OR instagram OR snapchat OR blog OR tumblr OR myspace) AND (engag* OR participat* OR involv* OR partner* OR codesign* OR coprod* OR co-design* OR co-prod* OR quality improvement) AND (hospital* OR health service* OR medical service* OR policy OR policies)	2004 – current (16 August 2022)

Table S3. Screening template:phenomenon of interest: Social media use to influence health service change or conduct quality improvement activities

CATEGORY	Criteria	Y	N	Unclear/Notes
**POPULATION**	**1. Does this study include participants who are health services users and/or health service providers?**<br><br>Rate as Y if the study includes participants who are:<br><br>a. Users or potential users of the health service (i.e., patients, consumer representatives, consumers with an acute or chronic condition, carers, family members, consumer organization member, community members, public)<br><br>AND/OR<br><br>b. Health service providers (i.e, health professionals, health service manager/administrator, health policy makers)<br><br>Rate as "N" if the study includes none of the above participants (e.g., academics, educators, researchers only)			
**CONCEPT**	**2. Can the online platforms in the study be considered 'social media'**<br><br>Rate as "Y" if the online platforms in the study allow all users (not just platform owners/operators) to create content which can be seen by other users of the platform. This may include public open platforms or private platforms requiring registration.<br>• Common public platforms are: Facebook, Twitter, MySpace, YouTube (providing comments aren't disabled), Snapchat, Instagram, Google+, Patient Opinion, Yelp.<br>• Common words/phrases in a platform description that may indicate a platform is social media include (but are not limited to): chat function, discussion board/thread, rate, review, comments, blog, moderator, community manager.<br><br>Rate as "N" if:			

	• the platform is a website only with no indication that two-way communication is occurring<br>• email is the only form of online communication (i.e., communication is not visible to all users)<br>• there is no use of social media (e.g., face to face, teleconference or videoconference meetings).			
	• **Are the participants:**<br>o **Involved in a quality improvement project or activity? AND/OR**<br>o **Trying to influence a change in health service or system design or the services being delivered?**<br><br>**Quality improvement** is defined as "the combined efforts of the workforce and others – including consumers, patients and their families, researchers, planners and educators – to make changes that will lead to better patient outcomes (health), better system performance (care) and better professional development."<br><br>Rate as "Y" if:<br>• The study describes an intention to change how health services or systems are designed or delivered through the involvement of the participants in quality improvement activities and/or other actions aimed at influencing change; OR<br>• The study describes a change/attempt at change that has occurred to health services or systems based on the actions or activities of the participants.<br><br>Note: Studies can still be included if the change or quality improvement activity has not been successful or is ongoing/incomplete.<br><br>Rate as "N" if:<br>• Social media is used for disease surveillance only without stated intended or actual change to current health service or system design/delivery (recommendations for change alone not sufficient).<br>• Social media is for health information dissemination only.<br>• Social media is for patient treatment/care/peer support within existing model of care and without intention to			

	influence changes to health service or quality improvement activities.<br>• Social media is used in health provider education or consumer education which doesn't have impacts on a how a health service designs or delivers existing services (for e.g., social media use in undergraduate health care provider education).<br>• Consumer/service provider engagement in research only without changes to the design/delivery of an existing health service or system.<br>• Social media use for research recruitment.			
	**3. Are the participants using social media as a communication tool for their quality improvement activity/attempt to influence health service/system change?**<br><br>Rate as "Y" if:<br>• Some or all of the communication about the quality improvement activities and/or health service/systems change happens via social media.<br><br>Rate as "N" if the group:<br>• Communication about the quality improvement activities and/or health service/systems change happens via other mediums (face to face, telephone, videoconference, email) even if the new/changed service is delivered via social media.			
**CONTEXT**	• **Is this study primary research, review research and/or eligible grey literature?**<br><br>Rate as "Y" if the study is:<br>• Original primary research or evaluation article (any methods) or secondary review research article (including systematic reviews, meta-analyses, meta-syntheses, narrative reviews, mixed-methods reviews, qualitative reviews and rapid reviews)<br>• Published as either peer reviewed academic literature or within the grey literature.<br><br>Rate as "N" if:<br>• The article is an opinion piece, commentary or editorial without original research.			

	• The article is an overview of research literature but doesn't describe review methods.<br>• The article promotes or encourages advocacy activities without accompanying research examining the outcomes or experiences of the advocacy activity being promoted.<br>• The article has no stated research method or analysis of the data collected through the research.			
	**4. Is this study in a healthcare or health policy setting?**<br><br>Rate as 'Y' if:<br>• The study is set in a hospital, health service, aged care, community health, primary health or government health department; OR<br>• The participants are attempting to influence changes to existing health services designed/delivered in a hospital, health service, aged care, community health, primary health or government (local, federal, state) health department.<br><br>Rate as 'N' if:<br>• The study is set in a university, educational, research or other non-healthcare setting and there is no attempt to influence changes to existing health services or policies.			

Note:To be INCLUDED a study must be rated yes (Y) for EACH criteria in EACH category

There are three different scenarios after you finish your assessment:

1. IF THE ARTICLE SCORED **Y** ON EACH OF THE ABOVE CRITERIA -> CLASSIFY AS **INCLUDE**
2. IFTHE ARTICLE SCORED **N** TO ANY OF THE ABOVE CRITERIA -> CLASSIFY AS **EXCLUDE**
3. If IT IS <u>UNCLEAR</u> WHETHER AN ARTICLE MEETS A PARTICULAR CRITERIA, BUT THE ARTICLE WOULD **OTHERWISE BE INCLUDED**, classify as **INCLUDE** but put a note in the 'notes' section: MORE INFORMATION REQUIRED ABOUT [INSERT RELEVANT CRITERIA]
4. IF IT IS <u>UNCLEAR</u> WHETHER AN ARTICLE MEETS A PARTICULAR CRITERIA, BUT THE ARTICLE WOULD **OTHERWISE BE EXCLUDED**, classify as **EXCLUDE**

Table S4. Frequency of themes and codes in included studies.

Themes	Number of studies (n(%)) reporting themes and codes
Reported benefits of social media use	Benefits reported in 51 studies (84%)<br><br>*Improves organisational communication*<br>Improves the efficiency of communication = 31 (51%)<br>Provides an additional channel for communication = 14 (23%)<br><br>*Builds relationships*<br>Facilitates collaborative relationships = 23 (38%)<br>Engages new audiences = 15 (25%)<br>Improves clinical practice = 6 (10%)<br><br>*Higher quality information*<br>Improves the quality of information gathered or shared = 18 (30%)<br>Facilitates high quality discussions = 6 (10%)<br><br>*Improves organisational culture and reputation*<br>Facilitates positive organisational culture change = 14 (23%)
Reported risks/limitations of social media use	Risks/limitations reported in 40 studies (66%)<br><br>*Limited or ineffective engagement*<br>Underutilisation by target audiences = 26 (43%)<br>Quality of discussion/information gathered not sufficient for QI purposes = 10 (16%)<br><br>*Limited evidence of effectiveness*<br>Unclear evidence of the benefits compared to traditional stakeholder engagement methods = 15 (25%)<br><br>*Direct harm to individuals and organisations*<br>Malicious, fake or negative messages and actions = 13 (21%)<br>Breaches of privacy and professional behaviour = 6 (10%)<br><br>*Challenges to strategic use*<br>Difficult to use strategically to achieve change = 6 (10%)<br>Difficult to evaluate = 4 (7%)
Reported barriers to social media use	Barriers reported in 27 studies (44%)<br><br>*Lack of access to and familiarity with social media*<br>Lack of resources and access = 15 (25%)<br>Lack of skills and confidence in using social media = 7 (11%)<br>Lack of familiarity with using social media for health or QI purposes = 4 (7%)<br><br>*Lack of organisational processes and support*<br>Issues with organisational culture and lack of executive support = 8 (13%)<br>Concerns about implementation and evaluation processes = 7 (11%)

	Concerns about how people behave online<br>Concerns about managing messages, people and interactions in a public forum = 8 (13%)<br>Concerns about privacy and professional behaviour = 5 (8%)<br>Unwillingness to share personal information online = 5 (8%)<br><br>*Problems with social media platforms*<br>Rapid changes in the social media environment = 2 (3%)<br>Poor platform usability = 2 (3%)
Reported enablers of social media use	Enablers reported in 44 studies (72%)<br><br>*Facilitating access and use for all stakeholders*<br>Making use of social media easier for target audiences = 20 (33%)<br>Organisational systems, processes, resourcing and partnership = 14 (23%)<br>Providing multiple ways to engage = 11 (18%)<br>Facilitating access to social media = 5 (8%)<br><br>*Making discussions safe*<br>Making discussions safe = 15 (25%)<br><br>*Providing high quality content and incentives*<br>Delivering engaging, trustworthy and targeted content = 15 (25%)<br>Users gaining benefits from participation = 8 (13%)<br><br>*Building a social media community*<br>Fostering connections between users in a community = 12 (20%)<br>Organisations promoting their use of social media = 5 (8%)
